# Alleviating staff stress in care homes for people with dementia: protocol for stepped-wedge cluster randomised trial to evaluate a web-based Mindfulness- Stress Reduction course

**DOI:** 10.1186/s12888-015-0703-7

**Published:** 2015-12-21

**Authors:** Christine Baker, Peter Huxley, Michael Dennis, Saiful Islam, Ian Russell

**Affiliations:** Swansea University Medical School, Swansea, UK; Centre for Mental Health and Society, Bangor University, Bangor, Gwynedd UK

**Keywords:** Dementia, Challenging behaviour, Care homes, Care staff, Stress, Mindfulness, Stepped-wedge cluster randomised trial

## Abstract

**Background:**

There has been continuing change in the nature of care homes in the UK with 80 % of residents now living with some form of dementia or memory problem. Caring in this environment can be complex, challenging and stressful for staff; this can affect the quality of care provided to residents, lead to staff strain and burnout, and increase sickness, absence and turnover rates. It is therefore important to find interventions to increase the wellbeing of staff that will not only benefit staff themselves but also residents and care providers. Mindfulness training is known to be effective in treating a variety of physical and mental health conditions.

**Methods and design:**

The study uses mixed methods centred on a stepped-wedge cluster randomised trial. Thirty care homes in Wales are implementing a brief web-based mindfulness training course, starting in random sequence. Four to ten consenting staff from each facility undertake the course and complete validated questionnaires at baseline and after eight and 20 weeks. We shall also interview a stratified sample of ten trained staff and analyse the transcripts thematically. The primary outcome is stress; secondary outcomes include job satisfaction, attitudes towards residents and sickness absence rates.

**Discussion:**

With increasing numbers of people living with dementia in care homes and causing stress in their carers, it is important to evaluate support strategies for staff. Mindfulness-based therapies may be of potential benefit and need detailed examination.

**Trial registration:**

ISRCTN registry. ISRCTN80487202. Registered 24 July 2013.

## Background

In 2000 Jennings [1] warned that one of the biggest challenges to health and welfare services over the next 50 years would be to improve the capacity to provide good care for people living with dementia (PLWD). Dementia has since become a challenge in both extent and impact. In 2014, around 835,000 people in the UK had a form of dementia; with an ageing population this number will increase [2].

More than 80 % of older people are now living in residential and nursing homes have some form of dementia or memory problem [3]. Consequently long-term care for older people is mainly about care for dementia [4]. Whilst there are increasing numbers of residents living with dementia, the number of care home places has fallen in the last few years [5]. Projections of future demand suggest that more than double the current number of care home places will be required by 2043 to maintain the current ratio of institutional to community services for dementia [5].

The role of care homes in the UK has been changing over the past few years. This transformation was highlighted in an independent review established to recommend how to improve the training and support of both healthcare assistants working in hospitals, and social care workers in care homes and people’s own homes [6].

This change in the nature of care homes has made considerable demands on the skills and training of their workforce. Providing care for PLWD has become a major industry for direct care providers, who are under pressure to ensure staff are suitably educated and trained to meet the needs of these residents. The UK Government is committed to improving dementia care by enhancing knowledge, skills, training and understanding of dementia amongst staff, as evidenced by the National Dementia Strategies in England [7], Northern Ireland [8], Scotland [9] and Wales [10]. However it may be difficult for care home providers to sustain a workforce that, whilst motivated to provide care under difficult conditions, may suffer work-related stress as a consequence. The mental health and wellbeing of staff may also affect staff turnover and sickness absence, and thus increase costs to providers.

In the UK in 2013–4, stress, depression or anxiety accounted for 39 % of all work-related illnesses losing a total of 11.3 million working days. The occupations that reported the highest prevalence of stress-related illness were health and social care associate professionals [11].

Nursing has long been considered one of the most stressful professions [12–14]. The main stressors include long hours, heavy workload, lack of influence within the workplace, insufficient resources, role ambiguity, experiences of aggression [15], the effects of death and dying [16], and lack of support and recognition from co-workers and management [17]. Yet dominant nursing philosophies emphasise a humanistic caring paradigm that requires them to develop profound interpersonal understanding and sensitivity to provide effective care [18]. This dilemma can also apply to staff working in care homes who have similar stressors, and are also poorly paid. An influential social care workforce study estimated that about 10 % of the care workforce in England received less than the national minimum wage [19]. Care staff may also have zero-hour contracts which oblige them to work all shifts offered, often without holiday or sick pay [20].

Care workers are crucial to the quality of care and quality of life of PLWD in care homes, and the relationship between residents and caregivers is central to this quality [21]. However caring for PLWD can be stressful and challenging for staff [22–24]. As well as the complex physical requirements, up to 40 % of staff time is spent managing challenging resident behaviour [23, 25, 26]. This behaviour occurs in at least 75 % of people with dementia and may become more severe over time [27]. This contributes to staff stress, strain [23, 24, 28] and potentially to staff turnover. Consequently, residents have staff who are less willing to help, low in optimism and show negative emotional responses to clients’ behaviour [29].

Work stress is described as “adverse reactions to excessive pressures or other types of demand” [30]. Although there is controversy about the mechanisms of occupational stress, the dominant models – demand–control [31] and effort-reward [32] - explain it as an imbalance between these factors. The resultant effects of stress on individuals are mediated by personal factors like age, experience, health and coping skills [33–35].

The National Institute for Health and Care Excellence (NICE) has published two public health guidelines relevant to mental health in the workplace - Managing Long Term Sickness and Incapacity [36] and Promoting Mental Wellbeing at Work [37] - and recommended more evidence-based research, focused not only on organisations but also on individuals [37].

Whilst there has been research on stress in families caring for relatives with dementia, there are few studies of stress in long-term care staff [38]. One study examined stress in staff caring for residents with dementia across long-term care facilities in the USA [22]. Results showed that stress was reported more often by those employed for less than 2 years even though their attitudes towards dementia were more positive. The authors also found job satisfaction to be higher amongst staff who had received more training in assessment or who practised person-centred care (PCC) [22].

A systematic review in the Netherlands [39] showed that PCC generally increased job satisfaction and personal accomplishment, and reduced job demands and emotional exhaustion: one study found that PCC reduced sickness absence and two found that it reduced stress. The authors argued for more research into care homes with longer follow-up periods [39].

Despite studies of sources and levels of stress amongst health care professionals, little is known about effective ways to moderate or eliminate these stressors [40]. A rigorous review of interventions for reducing occupational stress amongst health care workers found that only two of 14 trials were of high quality and concluded that more rigorous research is needed [41].

Mindfulness-based stress reduction (MBSR) is an intervention that has been reported to be beneficial in the treatment of many physical and mental health problems [42, 43]. It is effective in the stress management of healthy people and helps to increase empathy and self-compassion [44]. There is evidence to suggest that mindfulness practice is effective in reducing stress and decreasing burnout amongst healthcare professionals including nursing staff [45] and has a positive impact on job satisfaction [46]. However there is very little evidence of the use of MBSR by care staff and nurses in long term care settings and its effects.

MBSR is a psycho-educational programme that typically comprises eight weekly classroom-based sessions lasting about 2 h and a whole-day retreat towards the end. The programme includes taught meditation practices, and exploits the group format by sharing and discussing the challenges and experiences of incorporating mindfulness into daily lives and stressful situations. However we needed to enable staff to attend group sessions despite geography, time pressures and the costs of attending a group course, which range between £200 and £350 per person [47].

Alternatives to the traditional method of teaching MBSR include synthesising the main elements, thus reducing the length of the programme [18]. Web-based MBSR training has also been developed; one study showed no difference in outcomes between MBSR participants who attended in person and those who trained online [48]. A feasibility study [47] and a randomised pilot study [49] both concluded that the online course was an accessible and acceptable way to reduce stress.

## Research objectives

### Primary objective

To evaluate the effectiveness of web-based mindfulness training in reducing staff stress in care homes with people living with dementia.

### Secondary objectives

To evaluate the effects of mindfulness training on: job satisfaction; staff attitudes to dementia; sickness absence; and its cost-effectiveness taking account of the time staff take to complete the course.

## Ethical approval

The Research Ethics Committee of the College of Human and Health Sciences of Swansea University approved this protocol. Informed written consent and permission to publish any direct quotes from interviews or focus groups will be obtained from each participant prior to the onset of the study.

## Design and methods

**Fig. 1:**
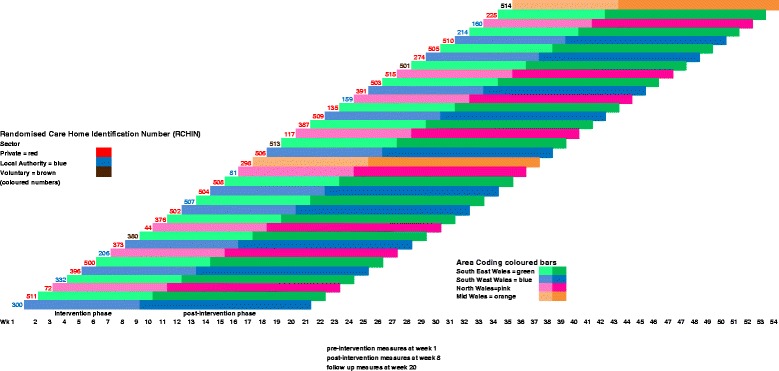
Stepped-Wedge Trial. Random sequence stratified by area and type of care home

The basic design is a stepped-wedge cluster randomised trial, also known as a randomised multiple interrupted time series. A trial of this nature rolls the chosen intervention out to clusters and their members in a random sequence over time [50]. Accordingly once every week or so we shall initiate a brief web-based mindfulness training programme for staff (who together form a ‘cluster’) in one of 30 participating care homes across Wales that are registered to care for PLWD, until all 30 have received the intervention. The Swansea Trials Unit (STU) will give each home an identification number to maintain anonymity and generate the random sequence stratified by area (South East, South West, North or Mid Wales) and type of care home (private, local authority or voluntary not for profit) (Fig. 1).

Though we know of no data on intra-cluster correlation coefficients (ICCCs) for stress in care home staff, our previous experience of cluster trials in care homes suggests that ICCCs are unlikely to exceed 0.05 [51]. So we estimate that if four consented staff in each of 30 trial homes report their stress levels before they start mindfulness training and eight and 20 weeks thereafter, the trial will have more than 80 % power to detect an improvement in stress equivalent to an effect size of 0.3 (generally regarded as a small effect).

In implementing this design, we shall conceal each home’s random start until 6 weeks before that date. We shall then inform managers by phone of the timing of their mindfulness training and arrange a visit to recruit and consent participating staff and show them how to access and use the online mindfulness training course.

## Recruitment

The Care & Social Services Inspectorate of Wales (CSSIW) provided a list of the 403 care homes in Wales registered to care for PLWD. We surveyed a random sample of 134 (33 %) of these. Of the 72 (54 0025) who responded, we selected a random sample of 35 to participate in the trial, thus permitting the immediate replacement of any of the 30 consenting homes who withdrew before starting.

We sent information sheets to care home managers to explain the trial and phoned interested managers to answer their questions and discuss the recruitment of staff to participate in the trial. We then attended staff meetings to explain mindfulness and gain voluntary participation.

## Inclusion and exclusion criteria

**Fig. 2:**
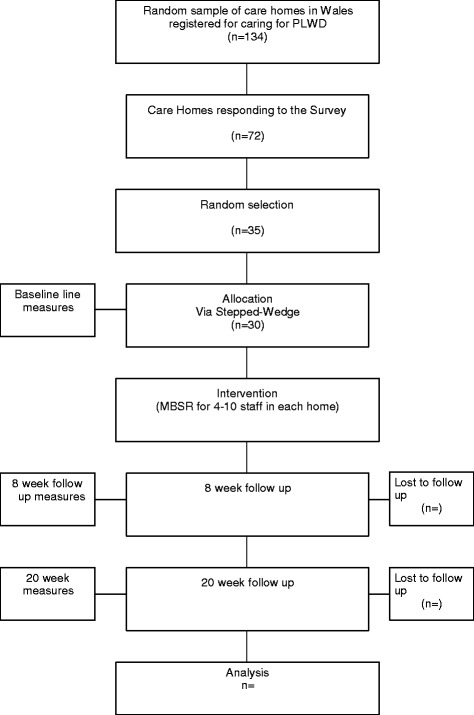
Flow Chart of recruitment process

Of the care homes registered with CSSIW to care for PLWD, we excluded those with fewer than 16 beds to yield enough staff for mindfulness training. In 30 consenting homes we approached managers and staff who provide direct care for residents. We sought to recruit at least four and at most ten staff, including at least one nurse where possible and at least two other care staff, to undertake mindfulness training and complete questionnaires before training and 8 and 20 weeks thereafter (Fig. 2).

## Intervention

Table 1 below summarises the web-based mindfulness training course, organised by the Mental Health Foundation through Wellmind Media [52]. It combines elements of both Mindfulness-Based Stress Reduction (MBSR) [53] and Mindfulness-Based Cognitive Therapy (MBCT) [54].

**Table 1:** Main programme components of mindfulness training

	Week 1	Week 2	Week 3	Week 4
	Introduction			
	Orientation			
	Stress Assessment			
Theme	Stepping out of	Reconnecting with	Working with	Mindfulness
	Automatic Pilot	Body and Breath	Difficulties	in Daily Life
Exercises	Routine activity	Mindful movement	Breathing Space	Breathing
	Mindful eating	Mindful Breathing	Sitting meditation	Space and action step
	Body Scan	Event Awareness	Stress Awareness	Activity
				Awareness
				Stress
				Strategies

The main features include:

- Ten sessions each lasting 30 min with videos and interactive exercises led by leading mindfulness trainers.
- Twelve assignments to practice in daily life with supporting emails.
- Five guided meditation audio downloads.
- Online tools for reviewing progress.
- A course completion certificate.
- An overview
- An aftercare pack sent by post including a printed guide to everyday mindfulness.

The course may be completed within 4 weeks but it can be done at a convenient pace as there is no limit to completion. It teaches formal meditation skills and informal techniques that can be incorporated into daily activities.

Participants can take breaks from the course and repeat any part at any time. If so they receive emails to remind them where they have reached. The software asks them to practice formal meditation exercises they have learned using the audio and video clips supplied, ideally every day.

Participants can monitor their progress in terms of stress, anxiety and depression, using measures intrinsic to the online course at the start and end, and at 1 month following completion. The measures include the Perceived Stress Scale [55], the Generalised Anxiety Disorder Assessment Scale (GAD-7) [56] and the Patient Health Questionnaire (PHQ-9) [57]. There is also an email address and telephone number for general and technical support.

### Measures used in the trial

The demographic and related data we collect include age, gender, ethnic origin, education, work status, training in dementia care (type, length and recency), care experience (both length and number of previous posts, with and without residents with dementia), and type and size of current care home.

### Outcome measures

At baseline, 8 weeks and 20 weeks we use seven validated measures to explore stress, job satisfaction and attitudes to residents with dementia. We complement these with qualitative data, reported sickness absence and reported costs.

### Primary outcome measures **—** stress

1. The Work Stress Inventory [58] has studied stress in staff of long-term care facilities in the USA [22]. It estimates the average frequency of 45 stressors across six domains — events, caring for residents, working relationships, supervisory relationships, workload and the caring environment.
2. The Karasek Job Content Questionnaire [59] was designed to assess the effects of stressful jobs on the health of employees. It is well validated and widely used and has been used in several countries with different economic and social characteristics. It comprises four scales **—** decision latitude (comprising two subscales **—** skill discretion and decision authority), psychological demands, physical demands and social support (comprising two subscales –support from colleagues and supervisors). Job strain arises from the combination of high demands and low decision latitude. Generic strain arises from the combination of high demands, low decision latitude and low social support.

### Secondary outcome measures

#### 1: Job satisfaction

- Staff Experience of Working with Demented Residents [60] uses 21 five-point items to assess satisfaction across six domains — feedback, the care organisation, ones’ own expectations, resident contact, the expectations of others and the environment.
- Satisfaction with Job Facets [61] uses five seven-point items to measure job satisfaction – with the job, with co-workers, with the work itself, with the place of work, and with the resources including equipment and supervision.

#### 2: Attitudes to dementia

The Approaches to Dementia Questionnaire [62] aims to discover respondents’ attitudes towards PLWD. It uses 19 five-point items to yield a total score and 2 sub-scores. The Person-Centredness (PC) dimension comprises 11 items assessing how the respondent acknowledges and reacts to PLWD as unique individuals; for example Question 11 states ‘people with dementia need to feel respected, just like anybody else’. The hope dimension comprises eight items and assesses whether the respondent is optimistic or pessimistic towards the abilities and the future of people with dementia; for example Question 3 states ‘there is no hope for people with dementia’. The scale is both consistent (Cronbach’s alpha = 0.83) and reliable (test-retest reliability = 0.76) [48].

#### 3: Health interference with work

The Stanford Presenteeism Scale 6 (SPS6) [63] uses six five-point items to measure the respondent’s “presenteeism”, defined as “ability to concentrate on and accomplish work despite health problem(s)” [60].

#### 4: Physical and mental wellbeing

The Short Form Health Survey (SF-12) [64] enables respondents to report their perceived wellbeing through eight concepts – physical functioning, role functioning physical, bodily pain, general health, vitality, social functioning, role functioning emotional, and mental health. These yield two meta-scores – Physical Component Summary (PCS) and Mental Component Summary (MCS) – each with a range of 0 to 100, and a mean of 50 and a standard deviation of 10 in a representative sample of the US population.

#### 5: Qualitative data

We want to explore the subjective experience of staff working in a care home in terms of stress and coping. As the web-based mindfulness programme is a novel way of reducing staff stress in this setting, we shall also ask participants about their experience of undertaking the training. To collect these qualitative data we shall conduct one-to-one semi-structured interviews with a sample of ten staff who consented to participate in the mindfulness training, stratified by the number of weeks they completed. This method will allow the participants to speak for themselves, complement the quantitative questions, and raise issues that may have not been previously considered. Table 2 shows the interview schedule: the first section covers questions about stress and coping in the care home; and the second asks questions about the web-based course itself.

**Table 2:** Qualitative interview schedule

A. Stress and coping in the care home
1 What do you think are the issues that cause you stress at work?
2 What sort of personal feelings do you have about working in a care home?
3 If you had any negative feelings, how would you have normally dealt with them?
4 Do you think that this was effective?
B. Experience of the Be Mindful course.
5 How did you feel about the course as a whole? (access, instructions, length of time taken, time negotiated with the manager to do the course)
6 What about the content of the course? Is there a specific type of practice that you feel benefited you most?
7 Did you personally feel any changes in your stress, anxiety or depression as a result of what you practiced or learned from the mindfulness course?
8 Do you think that this practice would help to reduce sickness/absence rates?
9 Are you still practicing some of the aspects you were taught on the course like mindfulness breathing?
10 Would you recommend this course or anything similar to colleagues or friends who were complaining of stress, anxiety or depression? If so why and if not, why not?
11 If you could change anything about the course, what would you suggest?
12 Is there anything else you would like to add?

We shall record the interviews digitally and transcribe them verbatim. We shall use the Computer Aided Qualitative Data Analysis Software (CAQDAS), NVivo 10, for data management and thematic analysis. We shall illustrate findings by anonymised quotations showing how mindfulness training affected respondents’ feelings of stress and wellbeing.

#### 6: Sickness absence rates

To examine the effects of the course on sickness absence rates, we shall ask managers for data from 3 months prior to the intervention until the 20-week follow up

#### 7: Costs

We shall estimate the costs of the intervention from discussions with providers and staff. Specifically we shall ask participating staff to report their use of health and social care and then use published unit costs to translate their responses into costs. We shall compare the resulting marginal cost of mindfulness training with its marginal benefit derived from the SF-6D [54], a by-product of the SF-12. Thus we shall estimate the cost per Quality-adjusted life year (QALY) gained by mindfulness training, the evaluative criterion favoured by the (British) National Institute of Health & Care Excellence (NICE).

## Statistical analysis

We shall analyse changes in all outcome measures between baseline and follow-up at 8 or 20 weeks by analysis of covariance. As covariates, we shall use the pre-training values of the outcome measure under analysis and consider participants’ age, gender, ethnic origin, education and work status, and features of their training in dementia care, care experience and current care home.

We shall check the internal consistency of these outcome measures by Cronbach’s alpha, and explore relationships between them by multiple linear regression. Where individual items are missing from outcome measures, we shall follow the published instructions for imputation if any; otherwise we shall use the SPSS Missing Value module.

## Discussion

The increasing number of people living with dementia (PLWD) in care homes and the need to protect these residents’ quality of life require us all to consider the wellbeing of staff. Very few studies have investigated stress in staff who care for PLWD in care homes. We know little about effective ways to ameliorate this stress. This trial will extend our knowledge by evaluating whether online mindfulness training reduces stress and improves job satisfaction and attitudes to dementia.

The advantage of using online training in this environment is that staff can access the course when convenient and at their own pace, and immediately use techniques learned in their practice. The trial participants include managers, qualified nurses, senior care workers and support staff with heterogeneous training, responsibilities and work patterns. Hence we plan to examine factors that influence stress and the effects of mindfulness training.

A major strength of the study is that the data come from a nationally representative random sample of care facilities for PLWD. In addition we are using mixed methods. Quantitative methods are valuable and have been used in several studies to evaluate the effects of MBSR in both clinical and non-clinical contexts. However those studies neglected subjective experiences of participants. Hence our use of qualitative interviewing will yield additional information and insights into the challenges of delivering MBSR to this population.

## Limitations and challenges

We are conscious that research in care homes is a complex undertaking which will require us to take account of the needs of residents, relatives and staff; and that participation in research may not be a priority for managers and staff who have high workloads [65].

The MBSR course requires a quiet room to listen to instructions and learn meditation practices. Participating staff also need access both to computers and the internet within their care homes. Time for undertaking MBSR training, and for completing questionnaires, also needs negotiation. We have addressed these issues in consultation with care home managers.

The web-based course is designed to take 4 weeks but staff can pause and restart at any time, so we shall not expect all staff to complete the course within the defined study period of 8 weeks. We shall therefore analyse whether the effectiveness of training is associated with participants’ rate of progress through the course.

We recognise that managers may be cautious about divulging information about sickness absence rates. We shall try to overcome this reluctance by spending time with staff to gain trust and confidence.

Despite these challenges, the result of the study will provide valuable evidence whether MBSR is an effective and cost-effective intervention in this organisational setting. If web-based mindfulness training proves effective in care homes, it would be important to consider whether to extend this training to informal and formal carers of PLWD across primary and secondary care who prefer web-based training or cannot attend traditional mindfulness courses.

## Abbreviations

CSSIW: Care and Social Services Inspectorate for Wales
MBCT: Mindfulness-Based Cognitive Therapy
MBSR: Mindfulness- Based Stress Reduction
MCS: SF-12 Mental Component Summary
NICE: (British) National Institute of Health & Care Excellence
NIHR: National Institute for Health and Care Excellence
PC: Person centredness
PCC: Person-centred care
PCS: SF-12 Physical Component Summary
PLWD: People living with dementia
QUALY: Quality-adjusted life year
STU: Swansea Trials Unit
